# Thiazolidinedione Use in Individuals With Type 2 Diabetes and Chronic Obstructive Pulmonary Disease

**DOI:** 10.3389/fmed.2021.729518

**Published:** 2021-12-09

**Authors:** Fu-Shun Yen, James Cheng-Chung Wei, Yu-Cih Yang, Chih-Cheng Hsu, Chii-Min Hwu

**Affiliations:** ^1^Dr. Yen's Clinic, Taoyuan, Taiwan; ^2^Department of Allergy, Immunology and Rheumatology, Chung Shan Medical University Hospital, Taichung, Taiwan; ^3^Institute of Medicine, Chung Shan Medical University, Taichung, Taiwan; ^4^Graduate Institute of Integrated Medicine, China Medical University, Taichung, Taiwan; ^5^Management Office for Health Data, China Medical University Hospital, Taichung, Taiwan; ^6^College of Medicine, China Medical University, Taichung, Taiwan; ^7^Institute of Population Health Sciences, National Health Research Institutes, Zhunan, Taiwan; ^8^Department of Health Services Administration, China Medical University, Taichung, Taiwan; ^9^Department of Family Medicine, Min-Sheng General Hospital, Taoyuan, Taiwan; ^10^Faculty of Medicine, National Yang-Ming University School of Medicine, Taipei, Taiwan; ^11^Section of Endocrinology and Metabolism, Department of Medicine, Taipei Veterans General Hospital, Taipei, Taiwan

**Keywords:** mortality, stroke, coronary artery disease, heart failure, non-invasive positive pressure ventilation, invasive mechanical ventilation, bacterial pneumonia, lung cancer

## Abstract

Few studies have investigated the effects of various antidiabetic agents on individuals with both type 2 diabetes mellitus (T2DM) and Chronic obstructive pulmonary disease (COPD). This study compared mortality, cardiovascular events and respiratory outcomes in individuals with both T2DM and COPD taking TZD vs. those not taking TZD. From Taiwan's National Health Insurance Research Database, 12 856 propensity-score-matched TZD users and non-users were selected between January 1, 2000, and December 31, 2012. Cox proportional hazards models were used to calculate the risks of investigated outcomes. Compared with non-use of TZD, the adjusted hazard ratios (95% CI) of TZD use were stroke 1.63 (1.21–2.18), coronary artery disease 1.55 (1.15–2.10), heart failure 1.61 (1.06–2.46), non-invasive positive pressure ventilation 1.82 (1.46–2.27), invasive mechanical ventilation 1.23 (1.09–1.37), bacterial pneumonia 1.55 (1.42–1.70), and lung cancer 1.71 (1.32–2.22), respectively. The stratified analysis disclosed that rosiglitazone, not pioglitazone, was associated with significantly higher risk of major cardiovascular events than TZD non-users. In patients with concomitant T2DM and COPD, TZD use was associated with higher risks of cardiovascular events, ventilation use, pneumonia, and lung cancer. Use of TZD in these patients should be supported by monitoring for cardiovascular and respiratory complications.

## Introduction

Chronic obstructive pulmonary disease (COPD) involves persistent inflammation of the airways and pulmonary microvasculature, leading to partially reversible airflow limitation (1). Inadequate treatment of acute COPD exacerbation can lead to hospital admission, respiratory failure, and even death (2). Approximately 10.1% of people in the world have COPD, and the prevalence of COPD continues to rise; this may be due to increased smoking and air pollution (3). COPD is the fourth leading cause of death in the world, causing approximately 3.2 million deaths in 2017 (2).

COPD often coexists with type 2 diabetes mellitus (T2DM) perhaps due to common pathogenic factors (4). Additionally, due to inflammatory processes or the use of high-dose steroids for its treatment, COPD may contribute to the development of T2DM (4). T2DM reduces lung function, aggravates bacterial infection, accelerates COPD progression, and increases mortality risk (4). The 2020 GOLD reports emphasizing that COPD often coexists with other diseases, through the related mechanisms such as chronic inflammation and smoking, which may have significant influences on disease course (5). Therefore, patients with both COPD and T2DM must be appropriately and carefully managed.

Few studies have evaluated the antidiabetic management in individuals with coexisted T2DM and COPD (6). Metformin use has been reported to be associated with a lower mortality risk in these patients (7, 8); but the long-term results of other anti-diabetic medications for these patients have not been carefully analyzed (6). Considerable efforts have been made to find new therapies that target inflammation in COPD but with little success.

TZDs are the ligands of peroxisome proliferator-activated receptor-γ (PPAR-γ) and are used as insulin sensitizers in T2DM treatment. Their use in individuals with diabetes also reduces systemic inflammation (6). Animal studies have discovered that TZD can attenuate airway inflammation and lung injury (6). Clinical studies have also revealed that TZD was associated with lower risks of COPD exacerbations (9) and lung cancer (6). However, one meta-analysis indicated that TZD increased the risk of pneumonia or lower respiratory tract infection (10). Thus, the effects of TZD use on patients with COPD remain uncertain. We therefore hypothesize that subjects with COPD and T2DM who are treated with TZD would demonstrate reduced risk of COPD exacerbation, major adverse cardiac events, and mortality.

## Results

### Participants

**Figure 1:**
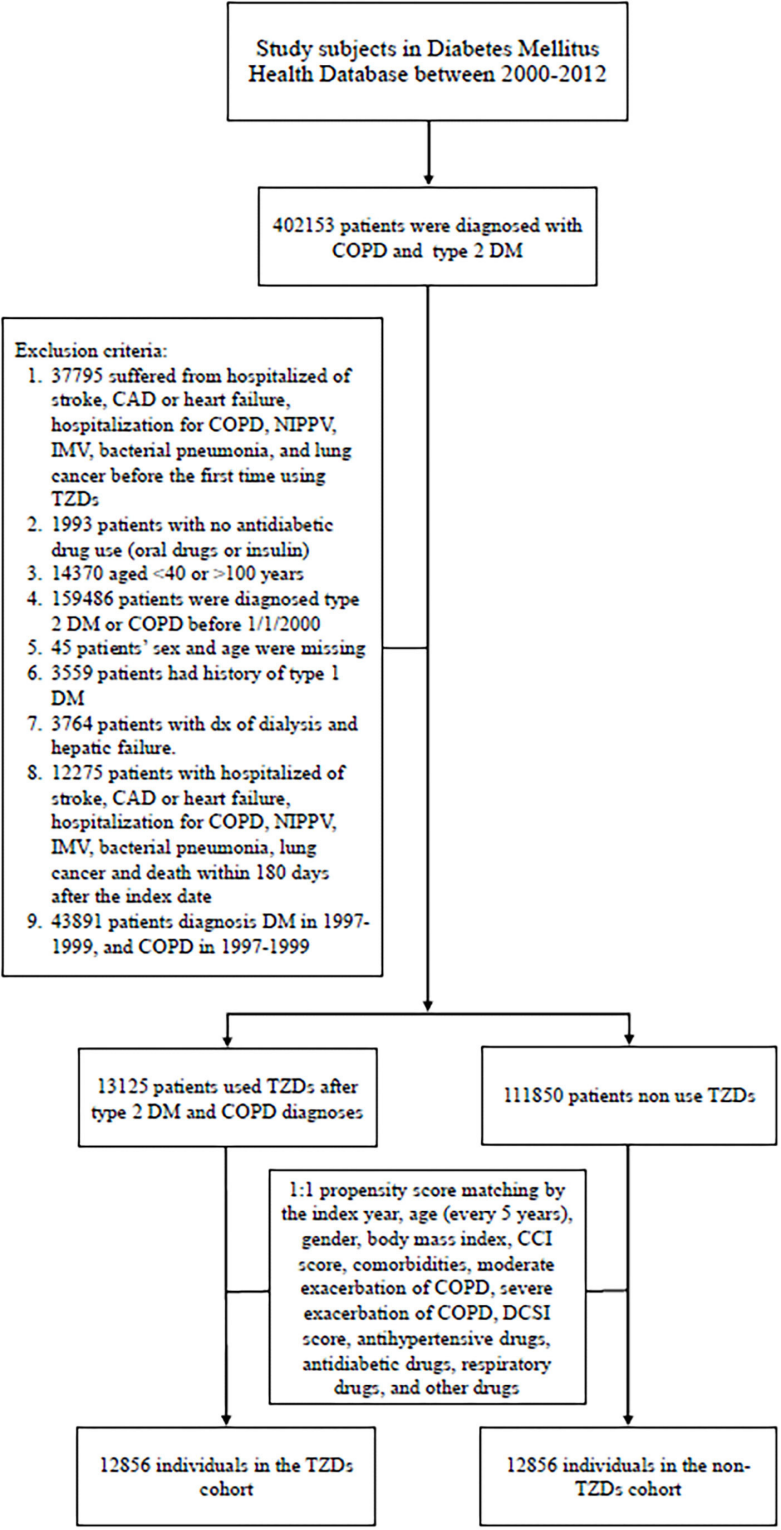
Patient selection flowchart.

Between January 1, 2000, and December 31, 2012, 402 153 patients were diagnosed as having T2DM and COPD. After applying the exclusion criteria, we identified 13 125 TZD users and 111 850 non-users during the study period. Figure 1 presented the patient selection flowchart.

Before propensity score matching, we observed some differences between TZD users and non-users (Table 1). After matching, 12 856 paired patients with T2DM and COPD were selected. In the matched cohorts, the mean (SD) age was 60.95 (9.96) years, and the mean T2DM duration was 8.81 (3.43) years.

**Table 1:** Patients' baseline clinical and demographic characteristics.

**Variable**	**Original population**				**SD^**§**^**	**P-S matching population**				**SD^**§**^**
	**TZD users**		**Non-users**			**TZD users**		**Non-users**		
	**(*****n*** **= 13,125)**		**(*****n*** **= 1,11,850)**			**(n = 12,856)**		**(*****n*** **= 12,856)**		
	***n***	**%**	***n***	**%**		***n***	**%**	***n***	**%**	
**Gender**										
Female	5,963	45.4	51,649	46.2	0.015	5,839	45.4	5,759	44.8	0.013
Male	7,162	54.6	60,201	53.8	0.015	7,017	54.6	7,097	55.2	0.013
**Age**										
40–64	8,011	61.1	61,706	55.2	0.119	7,792	60.6	9,115	70.9	0.218
≧65	5,114	38.9	50,144	44.8	0.119	5,064	39.4	3,741	29.1	0.218
**Obesity**	363	2.77	2,615	2.34	0.027	352	2.74	359	2.79	0.003
**Comorbidity**										
CAD	5,169	39.4	38,085	34.1	0.111	4,980	38.7	4,896	38.1	0.013
Stroke	2,712	20.7	22,864	20.4	0.005	2,618	20.3	2,627	20.4	0.002
Heart failure	968	7.38	7,335	6.56	0.032	935	7.27	919	7.15	0.005
PAOD	1,248	9.51	8,683	7.76	0.062	1,183	9.2	1,137	8.84	0.012
Atrial fibrillation	235	1.79	1,977	1.77	0.002	229	1.78	228	1.77	0.001
**Charlson comorbidity index**										
0	7,231	55.1	72,665	65	0.203	7,113	55.3	8,463	65.8	0.216
1	2,633	20	16,140	14.4	0.149	2,568	20.0	1,950	15.2	0.127
≧2	3,261	24.9	23,045	20.6	0.101	3,175	24.7	2,443	19.0	0.138
**Moderate exacerbation of COPD**										
0–1/previous year	10,997	83.8	93,330	83.4	0.009	10,761	83.7	10,777	83.8	0.003
≧2/previous year	2,128	16.2	18,520	16.6	0.009	2,095	16.3	2,079	16.2	0.003
**Severe exacerbation of COPD**										
0	13,052	99.4	1,11,146	99.4	0.01	12,875	99.4	12,783	99.4	0.002
≧1/previous year	73	0.56	704	0.63	0.01	71	0.55	73	0.57	0.002
**DCSI score**										
0	9,696	73.9	87653	78.4	0.106	9,519	74.0	9,559	74.3	0.007
1	1,814	13.8	11736	10.5	0.102	1,764	13.7	1,747	13.6	0.004
≧2	1,615	12.3	12461	11.1	0.036	1,573	12.2	1,550	12.1	0.005
**Respiratory drugs**										
β2 inhalants	4,307	32.8	42175	37.7	0.103	4,243	33.0	4,218	32.8	0.004
Anticholinergic inhalants	2,062	15.7	23201	20.7	0.131	2,041	15.8	2,022	15.7	0.004
Corticosteroid inhalants	1,301	9.91	11879	10.6	0.023	1,286	10.0	1,295	10.0	0.002
Oral corticosteroid	12,480	95.1	105986	94.7	0.015	12,223	95.1	12,194	94.8	0.01
Methylxanthine	12,188	92.8	103052	92.1	0.028	11,933	92.8	11,879	92.4	0.016
**Antidiabetic drugs**										
Metformin	12,493	95.1	58786	52.5	1.11	12,224	95.1	12,174	94.7	0.018
Sulfonylureas	12,241	93.2	49338	44.1	1.25	11,972	93.1	11,915	92.7	0.017
DPP4-inhibitors	5,338	40.7	10381	9.28	0.778	5,075	39.5	5,121	39.8	0.007
Insulin	5,906	45.0	20482	18.3	0.599	5,688	44.2	5,537	43.1	0.024
**Cardiovascular drugs**										
ACEI/ARB	8,945	68.1	61284	54.8	0.277	8,727	67.8	8,766	68.2	0.007
β-blockers	5,162	39.3	48366	43.2	0.08	5,100	39.7	5,126	39.9	0.004
CCBs	7,236	55.1	63298	56.6	0.029	7,141	55.6	7,135	55.5	0.001
Diuretics	3,725	28.4	32124	28.7	0.008	3,677	28.6	3,661	28.5	0.003
Statin	8,239	62.7	48089	42.9	0.404	8,005	62.3	7,989	62.1	0.003
Aspirin	5,560	42.3	43346	38.7	0.074	5,465	42.5	5,512	42.9	0.007
DM duration, mean(SD)	9.09 (3.27)		6.90 (3.80)		0.618	9.04 (3.27)		8.58 (3.59)		0.132

*SD, standardized difference; P-S, propensity score; TZD, thiazolidinedione; CAD, coronary artery disease; PAOD, peripheral arterial occlusion disease; COPD, chronic obstructive pulmonary disease; DCSI, Diabetes Complications Severity Index; DPP-4 inhibitors, dipeptidyl peptidase-4 inhibitors; ACEI, angiotensin-converting enzyme inhibitor; ARB, angiotensin receptor blocker; CCBs, Calcium-channel blockers*.

§ *A standardized mean difference of ≤0.10 indicates a negligible difference between the 2 cohorts*.

### Main Outcomes

In the matched cohorts, 17 (0.13%) TZD users and 19 (0.15%) non-users died during follow-up (incidence rate: 0.29 vs. 0.20 per 1000 patient-years). The multivariable-adjusted hazard ratio (aHR) of TZD users compared with non-users was 1.57 (95% CI = 0.78–3.15; Table 2).

**Table 2:** Outcomes of thiazolidinedione users and non-users.

	**TZD non-users**			**TZD users**			**Crude**		Adjusted^*^	
	**Events**	**PY**	**IR**	**Events**	**PY**	**IR**	**HR (95% CI)**	***p*** **value**	**HR (95% CI)**	***p*** **value**
Death	19	95,547	0.20	17	57,686	0.29	1.74 (0.88–3.45)	0.1	1.57 (0.78–3.15)	0.19
MACE	101	94,933	1.06	96	57,220	1.68	1.28 (0.96–1.70)	0.08	1.30 (0.97–1.73)	0.07
Stroke	101	95,114	1.06	96	57,335	1.67	1.63 (1.23–2.18)	0.0007	1.63 (1.21–2.18)	0.001
Coronary artery disease	98	95,116	1.03	88	57,361	1.53	1.54 (1.14–2.07)	0.004	1.55 (1.15–2.10)	0.003
Heart failure	52	95,358	0.55	44	57,556	0.76	1.60 (1.06–2.43)	0.02	1.61 (1.06–2.46)	0.02
Hospitalization for COPD	627	93,609	6.70	433	56,719	7.63	1.20 (1.06–1.36)	0.003	1.02 (0.89–1.16)	0.74
NIPPV	181	95,346	1.90	180	57,434	3.13	2.02 (1.62–2.51)	<0.0001	1.82 (1.46–2.27)	<0.0001
IMV	762	94,673	8.05	556	57,112	9.74	1.33 (1.19–1.49)	<0.0001	1.23 (1.09–1.37)	0.0004
Bacterial pneumonia	1205	92,596	13.01	1040	55,505	18.74	1.59 (1.46–1.74)	<0.0001	1.55 (1.42–1.70)	<0.0001
Lung cancer	127	95,380	1.33	125	57,521	2.17	1.76 (1.36–2.28)	<0.0001	1.71 (1.32–2.22)	<0.0001

*TZD, thiazolidinedione; PY, person-years; IR, incidence rate, per 1000 person-years; HR, hazard ratio; MACE, major adverse cardiovascular events; COPD, chronic obstructive pulmonary disease; NIPPV, non-invasive positive pressure ventilation; IMV, invasive mechanical ventilation*.

* *Models adjusted by sex, age, obesity, Charlson Comorbidity Index, moderate exacerbation of COPD, severe exacerbation of COPD, DCSI score, DM duration, and medications listed in Table 1*.

**Figure 2:**
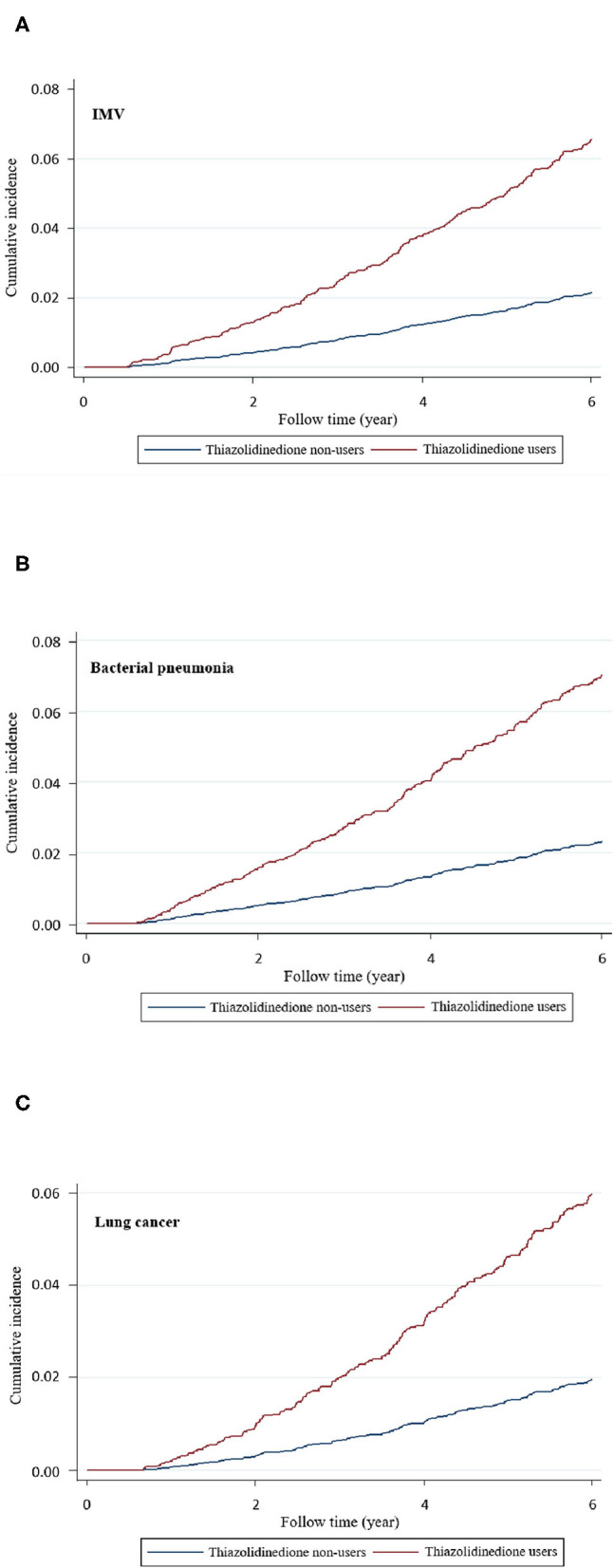
Cumulative incidence of **(A)** invasive mechanical ventilation (IMV), **(B)** bacterial pneumonia, and **(C)** lung cancer in thiazolidinedione users and nonusers.

As presented in Table 2, compared with non-users, TZD users had significantly higher risks of stroke (aHR 1.63, 95% CI 1.21–2.18), coronary artery disease (CAD; aHR 1.55, 95% CI 1.15–2.10), heart failure (aHR 1.61, 95% CI 1.06–2.46), non-invasive positive pressure ventilation (NIPPV; aHR 1.82, 95% CI 1.46–2.27), invasive mechanical ventilation (IMV; aHR 1.23, 95% CI 1.09–1.37), bacterial pneumonia (aHR 1.55, 95% CI 1.42–1.70), and lung cancer (aHR 1.71, 95% CI 1.32–2.22). The cumulative incidences of IMV, bacterial pneumonia, and lung cancer were higher in TZD users than in non-users (Figure 2).

### Additional Analyses

Supplementary Tables S1–S3 online presented the results of stratified analyses of IMV, bacterial pneumonia and lung cancer between TZD users and non-users. Higher risks in TZD users than in TZD non-users were observed in patients with the following characteristics: use of rosiglitazone, men, lower Charlson Comorbidity Index (CCI) or Diabetes Complication Severity Index (DCSI) scores, less COPD exacerbation, and use of medications. Supplementary Table S4 presented the results of stratified analyses of stroke, CAD and heart failure between TZD non-users, users, rosiglitazone and pioglitazone. Rosiglitazone users were associated with higher risks of stroke, CAD and heart failure. Supplementary Tables S5–S7 presented the results of dose response effects of TZD use in the risks of bacterial, pneumonia, IMV and lung cancer; which disclosed that most of cumulative durations and defined daily doses (DDDs) of TZD use had significantly higher aHRs than TZD no-use. Supplementary Table S8 presented the main outcomes after excluding patients with major cardiovascular events, lung cancer or death within 365 days after the index date, and adding smoking status as a covariate for propensity score matching; which revealed that TZD users were associated with significantly higher risks of NIPPV 1.49(1.20–1.86), IMV 1.17(1.05–1.32), bacterial pneumonia 1.58(1.44–1.73), and lung cancer 1.56(1.19–2.05) than TZD non-users.

## Discussion

Our study demonstrated that TZD use in patients with T2DM and COPD was associated with significantly higher risks of cardiovascular diseases, ventilation support, bacterial pneumonia, and lung cancer as compared with TZD non-users. Moreover, the results of stratified analysis revealed the association of TZD use with higher risks of IMV, bacterial pneumonia, and lung cancer in different subgroups of patients, with rosiglitazone use seeming to have higher risks than pioglitazone use.

Cardiovascular complications are the main cause of death in individuals with T2DM (11), and patients with COPD often develop cardiovascular diseases (12). One randomized trial demonstrated that in individuals with insulin resistance and stroke, pioglitazone use can reduce the risks of stroke or myocardial infarction compared with pioglitazone non-use (13). One cohort study using the Taiwan's National Health Insurance Database disclosed that TZD added on to metformin was associated with lower major cardiovascular risk when compared with sulfonylurea added to metformin (14). However, TZD use is generally associated with body weight increase and heart failure (15). In our study, compared with TZD non-use, TZD use was associated with significantly higher risks of stroke, CAD, and heart failure and a non-significantly higher risk of composite major adverse cardiovascular events (MACEs). The stratified analysis of our study disclosed that rosiglitazone, not pioglitazone, was associated with significantly higher risks of major cardiovascular events than TZD non-users. The different results between ours and the abovementioned two studies may be due to TZD use in different population (our study was for patients with T2DM and COPD, the randomized trial was for individuals with insulin resistance and stroke, the cohort study was for persons with T2DM and metformin failure). Taken together, the findings imply that individuals with T2DM and COPD taking rosiglitazone should be monitored for cardiovascular complications.

People with COPD have limited airway patency, mucus hypersecretion, and impaired pulmonary function, which increases their susceptibility to acute exacerbation or hospitalization (16). T2DM accelerates COPD progression and worsens its prognosis (4). One cohort study revealed that TZD use in patients with coexistent COPD and T2DM was associated with a small but significantly lower risk of COPD exacerbation (9). A disease risk score matched nested case–control study in Taiwan disclosed that current TZD use for more than 181 days yielded about 40% reduced risk of COPD exacerbation in patients with comorbid T2DM and COPD (17). By contrast, we found that TZD use in patients with T2DM and COPD was not significantly associated with the risk of COPD hospitalization, probably due to differences in the methods and demographics of these studies.

Acute exacerbations in patients with COPD are frequently accompanied by bacterial infections (2, 18), or necessitating systemic corticosteroids, both of which may increase the risk of pneumonia (19). T2DM impairs patients' immune responses and increases the risk of respiratory infections (4), which further enhances the propensity of patients with concomitant COPD and T2DM to develop bacterial pneumonia. A meta-analysis on TZD use in individuals with COPD demonstrated that TZD use may increase the risk of bacterial pneumonia and lower respiratory tract infection (10), which was consistent with our finding that TZD users had a higher risk of bacterial pneumonia than did non-users. The plausible mechanisms for this result may be that TZDs are PPAR-γ agonists, and TZD-mediated PPAR-γ activation has anti-inflammatory and immunomodulatory effects on the lungs (20). TZDs can also activate glucocorticoid receptors, increase glucocorticoid-like peptide secretion, and reduce host immune function (21, 22).

Oxygen therapy improves survival in patients with COPD with hypoxemia (19). Patients have hypoxemia with persistent hypercapnia benefit from non-invasive ventilation, but those who have hypercapnia with unstable condition require conventional mechanical ventilation (23). Our study revealed that TZD use might increase the risks of NIPPV and IMV. This may be because the higher risk of bacterial pneumonia and lung cancer associated with TZD use may increase the risks of hypoxia and respiratory failure.

Because most patients with COPD have a history of smoking, they are also more likely to develop lung cancer (24). Diabetes has been associated with a higher risk of lung cancer (6). TZDs bind to PPAR and have antitumor properties (6). Some studies have shown that TZDs may reduce the risk of lung cancer (6), whereas others have reported the opposite effect (25). Our study demonstrated that TZD use may increase the risk of lung cancers, and this result was persistent among different subgroups of patients with T2DM and COPD.

TZDs combined with DPP-4 inhibitors seemed to cause a higher risk [aHR 1.74 (1.28–2.36)] of IMV (Supplementary Table S1) than with the other antidiabetic agents. TZD use in patients with greater DSCI [DSCI≧2, aHR 2.46 (1.25–4.82)] and DPP-4 inhibitors [aHR 2.24 (1.18–4.25)] had a higher risk of lung cancer (Supplementary Table S3). Patients who took TZD combined with DPP-4 inhibitor may indicate suboptimal control of blood glucose. Patients with higher DSCI may indicate higher severity of T2DM. These may suggest that TZD use in patients with more severe T2DM, their worsening effects of IMV and lung cancer are greater. Besides, in patients with severe exacerbation of COPD, TZDs seem to have higher aHRs of IMV [3.06 (0.87–10.7)] and pneumonia [1.75 (0.49–6.26)]; which may indicate that in patients with more severe COPD, TZD use may confer worse effects of IMV and pneumonia.

In our cohort, rosiglitazone appeared to have higher risks of IMV, pneumonia, and lung cancer than pioglitazone. Rosiglitazone is a PPAR-γ agonist, but pioglitazone is both PPAR-α and -γ agonist (10); as a result, they have different anti-inflammatory, immunomodulatory, and antitumor effects. Compared with pioglitazone, rosiglitazone has more potent renal PPAR-γ agonistic effect (26) and a higher risk of cardiovascular diseases (27). Taken together, these findings may explain the higher risks of respiratory outcomes of rosiglitazone use in our study.

Diabetes can worsen the clinical course of COPD, including death risk (6). Clinical trials involving patients with T2DM have not revealed an effect of TZD use on mortality (28). Although our study revealed that TZD use may increase the risks of NIPPV, IMV, bacterial pneumonia, and lung cancer, it was not associated with an increased risk of COPD hospitalization or mortality.

This study has several limitations. First, the National Health Insurance Research Database (NHIRD) does not have complete information on family history, dietary patterns, and physical activity; accurate population data for alcohol drinking and smoking habits also are difficult to obtain from this database. The lack of these data may have influenced the results of TZD use and outcome analyses. However, we used propensity score matching to balance clinically relevant variables between the 2 groups to minimize the bias of the known confounding factors. Second, the NHIRD lacked biochemical and pulmonary function test results, precluding the calculation of COPD severity scores. Instead, we used clinical records to evaluate the number of moderate and severe exacerbations of COPD. Third, this study only included people with Chinese ethnicity; thus, the results may not be applied to other ethnicities. Finally, the retrospective study design precludes causal determination, matching, and investigation of underlying mechanism, and randomized controlled trials are warranted to verify our results.

In this nationwide cohort study, patients with T2DM and COPD using TZDs might be associated with higher risks of cardiovascular complications (mainly rosiglitazone), respiratory support, bacterial pneumonia, and lung cancer than did those not using TZDs. When prescribing TZDs, to these patients, cardiovascular (especially rosiglitazone) and respiratory complications should be closely monitored.

## Methods

### Study Population

Taiwan's National Health Insurance (NHI) program was implemented in 1995 and has included >99% of the 23 million Taiwan residents since 2000 (29). The NHIRD contains information on the insured's sex, age, residence, salary, drug prescriptions, medical procedures, and diagnosis according to the International Classification of Diseases, Ninth Revision, Clinical Modification (ICD-9-CM). The Longitudinal Cohort of Diabetes Patients (LHDB) is part of the NHIRD, which selected 120 000 newly diagnosed patients with T2DM yearly from 1999 to 2012, and their medical records from 1996 to 2013 are included in this dataset. All methods were performed in accordance with the Declaration of Helsinki. All identifiable information of patients and caregivers was encrypted before database release. This study was approved by the Research Ethics Committee of China Medical University and Hospital (CMUH104-REC2-115-CR4), and the need for informed consent was waived.

### Study Design

We recruited patients who had received diagnoses of T2DM and COPD in the LHDB between January 1, 2000, and December 31, 2012; they were followed up until December 31, 2013. The diagnosis of T2DM was considered using the ICD-9-CM code 250.xx for at least 2 outpatient visits or one hospitalization in 1 year. The primary diagnosis of COPD was considered using the ICD-9-CM codes 491, 492, and 496 for at least 2 outpatient claims or one hospitalization in 1 year. The algorithms for these diagnostic definitions have been validated with acceptable accuracy (30–32). Moderate COPD exacerbation was defined by the prescription of systemic corticosteroids or antibiotics, and it was managed in the outpatient setting; severe COPD exacerbation was considered in the event of hospitalization or an emergency room (ER) visit (33). Patients were excluded if they (1) were not aged between 40 and 100 years; (2) were not using antidiabetic drugs; (3) had missing age or sex data; (4) were diagnosed as having type 1 DM (250.1x), hepatic failure (570, 572.2. 572.4, 572.8), or undergoing dialysis (V56.0, V56.8, V45.1); (5) had stroke (430–438), CAD (410–414), heart failure (428), COPD hospitalization, NIPPV (93.90, 93.91), IMV (96.7), bacterial pneumonia (481, 486, 482.41, 482.8), or lung cancer (162.0, 162.2, 162.3, 162.4, 162.5, 162.8, 162.9) before the index date; (6) had admitted stroke, CAD or heart failure, COPD hospitalization, NIPPV, IMV, bacterial pneumonia, lung cancers, or death within 180 days after the index date to exclude latent diseases; or (7) had been diagnosed as having T2DM or COPD before January 1, 2000, to exclude prevalent diseases.

### Procedures

**Figure 3:**
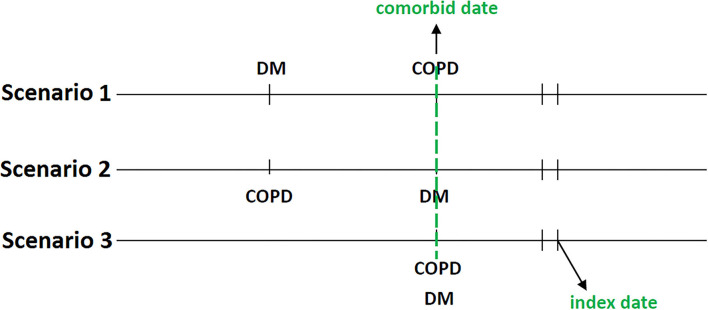
The concurrent diagnosis of T2DM and COPD was defined as the comorbid date. The first date of TZD use after the comorbid date was the index date.

The second date of the concurrent diagnosis of T2DM and COPD was defined as the comorbid date (Figure 3). Patients who had used TZDs for at least 28 days after the comorbid date were defined as TZD users, whereas those who were not prescribed TZDs during the study period were defined as TZD non-users. We regarded the first date of TZD use as the index date; the index dates of TZD non-users were randomly assigned after the comorbid date and within the follow-up period. Several variables were matched and adjusted in this study; they included age, sex, overweight [278.02, 783.1, V85.2], obesity [278.00, V77.8, 649.1, V85.3], severe obesity [278.01, 649.2, V45.86, V85.4]), comorbidities (CAD, stroke, heart failure, peripheral artery obstructive disease [PAOD; 440.0, 440.20, 440.21, 440.22, 440.23, 440.24, 440.3, 440.4, 443.9, 443.81, and 443.89], and atrial fibrillation [427.3]) diagnosed within 1 year before the index date, medications (respiratory drugs, antidiabetic medications, cardiovascular drugs), and T2DM duration. To accurately reflect the characteristics of our patients with T2DM and COPD, we used the CCI to assess patients' comorbidity profiles (34), the DCSI score (35) to evaluate T2DM severity, and the number of moderate or severe exacerbations of COPD to investigate the stability of COPD.

### Main Outcomes

All-cause mortality, MACEs, respiratory support, bacterial pneumonia, and lung cancers were the main outcomes of this study. Mortality was defined as discharge from hospital with the diagnosis of death. We calculated the incidence rates of composite MACEs and individual cardiovascular diseases, such as stroke, CAD, and heart failure. We scrutinized the incidence rates of hospitalization for COPD, NIPPV, IMV, bacterial pneumonia, and lung cancer to evaluate the progression of COPD.

### Statistical Analyses

We applied propensity score matching to optimize the comparability between TZD users and non-users (36). The propensity score was estimated for every patient by using a non-parsimonious multivariable logistic regression, with TZD use as the dependent variable. We included 36 clinically relevant covariates as independent variables (Table 1). The nearest-neighbor algorithm was used to constitute matched pairs, assuming that the standardized difference of ≤0.10 as a negligible difference between the study and control cohorts.

Crude and multivariable-adjusted Cox proportional hazards models were used to compare outcomes between TZD users and non-users. The results were presented as HRs and 95% CIs of TZD users compared with non-users. To calculate the mortality risk, we censored patients at the time of defined death or at the end of the study, whichever occurred first. For other investigated outcomes, we censored patients on the date of death, the date of respective outcomes, or at the end of the follow-up on December 31, 2013, whichever occurred first. We compared the cumulative incidence of IMV, bacterial pneumonia, and lung cancer over time between TZD users and non-users by using the Fine and Gray's subdistribution hazard model. We also performed stratified analyses in the risks of IMV, bacterial pneumonia, cardiovascular events and lung cancer among rosiglitazone, pioglitazone, and different subgroups of TZD users and non-users. To assess the dose response effects, we analyzed the risks of bacterial, pneumonia, IMV and lung cancer according to 3 cumulative equally distributed durations of TZD use (<100, 100–300, or >300 days) and 3 cumulative mean defined daily doses (DDDs) of TZD therapy (<5, 5–10, or >10 DDD/month), relative to non-use of TZD. DDD is a technical unit of measurement and is defined as the assumed average daily maintenance dose of a drug. The cumulative mean DDD was calculated by dividing the cumulative DDD by the duration of TZD use in months. According to the WHO, DDD for rosiglitazone is 6 mg, for pioglitazone is 30 mg, respectively. We have conducted a sensitivity analysis by excluding patients with major cardiovascular events, lung cancer or death within 365 days after the index date, and by adding smoking status (ICD-9-CM code: 305.1, 649.0, V15.82) as a covariate; we then went to compare the main outcomes of TZD use vs. TZD no-use using the multivariable-adjusted Cox proportional hazards model.

A two-tailed *P* value of < 0.05 was considered as significant. SAS (version 9.4; SAS Institute, Cary, NC, USA) was used for analyses.

## Data Availability Statement

The original contributions presented in the study are included in the article/Supplementary Material, further inquiries can be directed to the corresponding author/s.

## Ethics Statement

The studies involving human participants were reviewed and approved by Research Ethics Committee of China Medical University and Hospital (CMUH104-REC2-115-CR4). Written informed consent for participation was not required for this study in accordance with the national legislation and the institutional requirements.

## Author Contributions

F-SY: study concept and design, drafting of the manuscript, revision of the manuscript for crucial intellectual content, and study supervision. JW: revision of the manuscript for crucial intellectual content, technical or material support, and study supervision. Y-CY: data acquisition, analysis and interpretation, and statistical analysis. C-CH: analysis and interpretation of data, drafting of the manuscript, and revision of the manuscript for crucial intellectual content. C-MH: study concept and design, data acquisition, analysis and interpretation, drafting of the manuscript, revision of the manuscript for crucial intellectual content, statistical analysis, obtained funding, technical or material support, and study supervision.

## Funding

This study was supported in part by grants from the Taipei Veterans General Hospital (V105C-204 and V110C-175) and the Ministry of Science and Technology, R.O.C (MOST 110-2314-B-075-027-MY3), the Ministry of Health and Welfare Clinical Trial Center, Taiwan (MOHW109-TDU-B-212-114004), MOST Clinical Trial Consortium for Stroke, Taiwan (MOST 108-2321-B-039-003-), and Tseng-Lien Lin Foundation, Taichung, Taiwan. These funding agencies did not influence the study design, data collection and analysis, decision to publish, or preparation of the manuscript.

## Conflict of Interest

The authors declare that the research was conducted in the absence of any commercial or financial relationships that could be construed as a potential conflict of interest.

## Publisher's Note

All claims expressed in this article are solely those of the authors and do not necessarily represent those of their affiliated organizations, or those of the publisher, the editors and the reviewers. Any product that may be evaluated in this article, or claim that may be made by its manufacturer, is not guaranteed or endorsed by the publisher.
