# Supplementary material for: Thiazolidinedione Use in Individuals With Type 2 Diabetes and Chronic Obstructive Pulmonary Disease
Source: Front Med (Lausanne). 2021 Dec 9;8:729518. doi: 10.3389/fmed.2021.729518 (PMC8695877; doi:10.3389/fmed.2021.729518)
Supplement: Supplementary file 1 [file Data_Sheet_1.docx]

Supplementary Material

**Supplementary Table 1.** Hazard ratios and 95% CIs of IMV associated with thiazolidinedione use and nonuse stratified by age, sex, comorbidities, and medications.

| **Variable** | **TZD** | | | | | | **Crude HR (95% CI)** | **p-Value** | **Adjusted HR (95% CI)** | **p-Value** |
| --- | --- | --- | --- | --- | --- | --- | --- | --- | --- | --- |
|  | **No** | | | **Yes** | | |  |  |  |  |
|  | **Event** | **Person Year** | **IR** | **Event** | **Person Year** | **IR** |  |  |  |  |
| Overall | 762 | 94673 | 8.05 | 556 | 57112 | 9.74 | 1.33 (1.19-1.49) | <0.0001 | 1.23 (1.09-1.37) | 0.0004 |
| Rosiglitazone | 762 | 94673 | 8.05 | 328 | 31046 | 10.56 | 1.38 (1.21-1.57) | <0.0001 | 1.29 (1.13-1.48) | 0.0001 |
| Pioglitazone | 762 | 94673 | 8.05 | 228 | 26066 | 8.75 | 1.27 (1.09-1.49) | 0.002 | 1.16 (0.99-1.36) | 0.06 |
| **Gender** | | | | | | | | | | |
| Female | 270 | 43679 | 6.18 | 177 | 26047 | 6.80 | 1.24 (1.02-1.51) | 0.02 | 1.09 (0.89-1.33) | 0.39 |
| Male | 492 | 50994 | 9.65 | 379 | 31065 | 12.20 | 1.38 (1.20-1.58) | <0.0001 | 1.30 (1.13-1.50) | 0.0002 |
| **Age** | | | | | | | | | | |
| 40-64 | 374 | 69839 | 5.36 | 235 | 36533 | 6.43 | 1.32 (1.12-1.57) | 0.001 | 1.34 (1.13-1.58) | 0.0007 |
| ≧65 | 388 | 24834 | 15.62 | 321 | 20579 | 15.60 | 1.13 (0.97-1.32) | 0.1 | 1.18 (1.01-1.37) | 0.03 |
| **Comorbidity** | | | | | | | | | | |
| CAD | 321 | 34540 | 9.29 | 226 | 21067 | 10.73 | 1.30 (1.09-1.56) | 0.002 | 1.15 (0.96-1.38) | 0.11 |
| Stroke | 266 | 16837 | 15.80 | 158 | 10616 | 14.88 | 0.97 (0.79-1.19) | 0.8 | 0.92 (0.75-1.13) | 0.44 |
| Heart failure | 83 | 5950 | 13.95 | 55 | 3706 | 14.84 | 1.18 (0.83-1.68) | 0.34 | 0.91 (0.63-1.31) | 0.62 |
| PAOD | 83 | 7253 | 11.44 | 44 | 4561 | 9.65 | 0.87 (0.60-1.27) | 0.49 | 1.00 (0.68-1.48) | 0.98 |
| Atrial fibrillation | 20 | 1369 | 14.61 | 17 | 960 | 17.71 | 1.46 (0.74-2.88) | 0.27 | 1.16 (0.54-2.50) | 0.69 |
| **Charlson comorbidity index** | | | | | | | | | | |
| 0 | 357 | 66602 | 5.36 | 207 | 33940 | 6.10 | 1.34 (1.12-1.60) | 0.001 | 1.28 (1.07-1.53) | 0.007 |
| 1 | 158 | 13238 | 11.94 | 109 | 10567 | 10.32 | 1.03 (0.79-1.32) | 0.82 | 1.06 (0.81-1.37) | 0.65 |
| ≧2 | 247 | 14833 | 16.65 | 240 | 12605 | 19.04 | 1.17 (0.98-1.41) | 0.08 | 1.29 (1.07-1.56) | 0.007 |
| **Moderate exacerbation of COPD** | | | | | | | | | | |
| 0-1/previous year | 732 | 79103 | 9.25 | 534 | 47295 | 11.29 | 1.35 (1.20-1.51) | <0.0001 | 1.23 (1.10-1.39) | 0.0003 |
| ≧2/previous year | 30 | 15570 | 1.93 | 22 | 9817 | 2.24 | 1.16 (0.66-2.05) | 0.59 | 1.14 (0.63-2.09) | 0.65 |
| Severe exacerbation of COPD |  |  |  |  |  |  |  |  |  |  |
| 0 | 752 | 94111 | 7.99 | 546 | 56779 | 9.62 | 1.33 (1.18-1.49) | <0.0001 | 1.22 (1.08-1.37) | 0.0007 |
| ≧1/previous year | 10 | 562 | 17.79 | 10 | 333 | 30.03 | 1.83 (0.74-4.51) | 0.18 | 3.06 (0.87-10.7) | 0.07 |
| **DCSI score** | | | | | | | | | | |
| 0 | 483 | 69501 | 6.95 | 364 | 41806 | 8.71 | 1.40 (1.22-1.61) | <0.0001 | 1.24 (1.07-1.43) | 0.002 |
| 1 | 118 | 13931 | 8.47 | 85 | 8262 | 10.29 | 1.28 (0.95-1.70) | 0.09 | 1.21 (0.90-1.62) | 0.2 |
| ≧2 | 161 | 11241 | 14.32 | 107 | 7044 | 15.19 | 1.14 (0.89-1.47) | 0.28 | 1.15 (0.89-1.50) | 0.26 |
| **Respiratory drugs** | | | | | | | | | | |
| β2bronchodilators inhalants | 565 | 30380 | 18.60 | 419 | 19070 | 21.97 | 1.32 (1.16-1.51) | <0.0001 | 1.27 (1.11-1.45) | 0.0004 |
| Anticholinergic inhalants | 386 | 14153 | 27.27 | 286 | 9371 | 30.52 | 1.23 (1.05-1.44) | 0.009 | 1.25 (1.06-1.47) | 0.005 |
| Corticosteroid inhalants | 117 | 10028 | 11.67 | 72 | 5969 | 12.06 | 1.15 (0.85-1.56) | 0.36 | 1.01 (0.73-1.38) | 0.93 |
| Oral systemic corticosteroid | 739 | 90169 | 8.20 | 544 | 54216 | 10.03 | 1.35 (1.21-1.52) | <0.0001 | 1.24 (1.10-1.39) | 0.0002 |
| Methylxanthine | 711 | 87909 | 8.09 | 514 | 53063 | 9.69 | 1.32 (1.17-1.48) | <0.0001 | 1.20 (1.06-1.35) | 0.002 |
| **Antidiabetic drugs** | | | | | | | | | | |
| Metformin | 686 | 90086 | 7.61 | 514 | 54497 | 9.43 | 1.37 (1.22-1.55) | <0.0001 | 1.26 (1.12-1.42) | 0.0001 |
| Sulfonylureas | 712 | 88396 | 8.05 | 527 | 53568 | 9.84 | 1.36 (1.21-1.52) | <0.0001 | 1.24 (1.10-1.39) | 0.0004 |
| DPP4-inhibitors | 103 | 39206 | 2.63 | 93 | 23731 | 3.92 | 2.05 (1.52-2.75) | <0.0001 | 1.74 (1.28-2.36) | 0.0004 |
| Insulin | 463 | 40822 | 11.34 | 361 | 27228 | 13.26 | 1.28 (1.11-1.47) | 0.0006 | 1.24 (1.08-1.44) | 0.002 |
| **Cardiovascular drugs** | | | | | | | | | | |
| ACEI/ARB | 485 | 67103 | 7.23 | 361 | 41509 | 8.70 | 1.39 (1.21-1.61) | <0.0001 | 1.21 (1.05-1.40) | 0.007 |
| β-blockers | 261 | 42019 | 6.21 | 220 | 25759 | 8.54 | 1.68 (1.39-2.03) | <0.0001 | 1.43 (1.18-1.73) | 0.0002 |
| Calcium-channel blockers | 427 | 56302 | 7.58 | 326 | 35108 | 9.29 | 1.43 (1.23-1.66) | <0.0001 | 1.30 (1.12-1.51) | 0.0006 |
| Diuretics | 277 | 29898 | 9.26 | 231 | 19567 | 11.81 | 1.43 (1.19-1.71) | <0.0001 | 1.29 (1.07-1.55) | 0.006 |
| Statin | 283 | 16968 | 16.68 | 232 | 38200 | 6.07 | 1.66 (1.39-2.00) | <0.0001 | 1.35 (1.12-1.63) | 0.001 |
| Aspirin | 325 | 44132 | 7.36 | 253 | 27969 | 9.05 | 1.41 (1.19-1.67) | <0.0001 | 1.18 (0.99-1.41) | 0.05 |

TZD, Thiazolidinedione; PY, person-years; IR, incidence rate, per 1000 person-years; HR, hazard ratio; CAD, coronary artery disease; PAOD, peripheral arterial occlusion disease; COPD, chronic obstructive pulmonary disease; DPP-4 inhibitors, dipeptidyl peptidase-4 inhibitors; ACEI, angiotensin-converting enzyme inhibitor; ARB, angiotensin receptor blocker; DCSI, Diabetes Complications Severity Index. HR adjusted for sex, age, obesity, comorbidities, CCI score, DCSI score, and medication use.

**Supplementary Table 2.** Hazard ratios and 95% CIs of bacterial pneumonia associated with thiazolidinedione use and nonuse stratified by age, sex, comorbidities, and medications

| **Variable** | **TZD** | | | | | | **Crude HR (95%CI)** | **p-value** | **Adjusted HR (95%CI)** | **p-value** |
| --- | --- | --- | --- | --- | --- | --- | --- | --- | --- | --- |
|  | **No** | | | **Yes** | | |  |  |  |  |
|  | **Event** | **Person Year** | **IR** | **Event** | **Person Year** | **IR** |  |  |  |  |
| Overall | 1205 | 92596 | 13.01 | 1040 | 55505 | 18.74 | 1.59 (1.46-1.74) | <0.0001 | 1.55 (1.42-1.70) | <0.0001 |
| Rosiglitazone | 1205 | 92596 | 13.01 | 586 | 30012 | 19.53 | 1.58 (1.43-1.74) | <0.0001 | 1.58 (1.43-1.75) | <0.0001 |
| Pioglitazone | 1205 | 92596 | 13.01 | 454 | 25493 | 17.81 | 1.62 (1.44-1.81) | <0.0001 | 1.03 (1.03-1.04) | <0.0001 |
| **Gender** | | | | | | | | | | |
| Female | 411 | 42790 | 9.61 | 370 | 25410 | 14.56 | 1.58 (1.37-1.83) | <0.0001 | 1.56 (1.34-1.80) | <0.0001 |
| Male | 764 | 49806 | 15.34 | 670 | 30095 | 22.26 | 1.60 (1.43-1.78) | <0.0001 | 1.56 (1.40-1.74) | <0.0001 |
| **Age** | | | | | | | | | | |
| 40-64 | 601 | 68629 | 8.76 | 462 | 35677 | 12.95 | 1.65 (1.46-1.87) | <0.0001 | 1.79 (1.57-2.03) | <0.0001 |
| ≧65 | 604 | 23967 | 25.20 | 578 | 19828 | 29.15 | 1.30 (1.16-1.47) | <0.0001 | 1.45 (1.29-1.63) | <0.0001 |
| **Comorbidity** | | | | | | | | | | |
| CAD | 498 | 33707 | 14.77 | 410 | 20458 | 20.04 | 1.50 (1.31-1.72) | <0.0001 | 1.48 (1.29-1.70) | <0.0001 |
| Stroke | 370 | 16332 | 22.65 | 305 | 10225 | 29.83 | 1.37 (1.18-1.61) | <0.0001 | 1.41 (1.20-1.66) | <0.0001 |
| Heart failure | 115 | 5779 | 19.90 | 90 | 3571 | 25.20 | 1.34 (1.01-1.78) | 0.04 | 1.18 (0.88-1.60) | 0.25 |
| PAOD | 127 | 7101 | 17.88 | 94 | 4402 | 21.35 | 1.25 (0.95-1.65) | 0.1 | 1.43 (1.07-1.90) | 0.01 |
| Atrial fibrillation | 31 | 1316 | 23.56 | 23 | 939 | 24.49 | 1.10 (0.63-1.91) | 0.73 | 0.91 (0.48-1.70) | 0.77 |
| **Charlson comorbidity index** | | | | | | | | | | |
| 0 | 592 | 65477 | 9.04 | 459 | 33093 | 13.87 | 1.77 (1.56-2.01) | <0.0001 | 1.78 (1.57-2.03) | <0.0001 |
| 1 | 260 | 12815 | 20.29 | 208 | 10282 | 20.23 | 1.22 (1.00-1.47) | 0.04 | 1.28 (1.05-1.55) | 0.01 |
| ≧2 | 353 | 14304 | 24.68 | 373 | 12130 | 30.75 | 1.27 (1.10-1.48) | 0.001 | 1.46 (1.25-1.70) | <0.0001 |
| **Moderate exacerbation of COPD** | | | | | | | | | | |
| 0-1/previous year | 1205 | 76990 | 15.65 | 1040 | 45665 | 22.77 | 1.61 (1.48-1.76) | <0.0001 | 1.55 (1.42-1.70) | <0.0001 |
| ≧2/previous year | 0 | 15606 | 0.00 | 0 | 9840 | 0.00 | -- | -- | -- | -- |
| **Severe exacerbation of COPD** | | | | | | | | | | |
| 0 | 1189 | 92065 | 12.91 | 1031 | 55190 | 18.68 | 1.60 (1.47-1.75) | <0.0001 | 1.56 (1.43-1.70) | <0.0001 |
| ≧1/previous year | 16 | 531 | 30.13 | 9 | 315 | 28.57 | 0.97 (0.42-2.24) | 0.95 | 1.75 (0.49-6.26) | 0.38 |
| **DCSI score** | | | | | | | | | | |
| 0 | 797 | 68073 | 11.71 | 728 | 40706 | 17.88 | 1.70 (1.53-1.89) | <0.0001 | 1.59 (1.43-1.77) | <0.0001 |
| 1 | 153 | 13699 | 11.17 | 133 | 8025 | 16.57 | 1.66 (1.30-2.11) | <0.0001 | 1.71 (1.33-2.20) | <0.0001 |
| ≧2 | 255 | 10823 | 23.56 | 179 | 6774 | 26.42 | 1.19 (0.97-1.44) | 0.08 | 1.30 (1.06-1.60) | 0.01 |
| **Respiratory drugs** | | | | | | | | | | |
| β2bronchodilators inhalants | 881 | 29002 | 30.38 | 759 | 18014 | 42.13 | 1.54 (1.39-1.70) | <0.0001 | 1.55 (1.40-1.71) | <0.0001 |
| Anticholinergic inhalants | 583 | 13221 | 44.10 | 489 | 8691 | 56.27 | 1.41 (1.25-1.60) | <0.0001 | 1.48 (1.30-1.68) | <0.0001 |
| Corticosteroid inhalants | 179 | 9676 | 18.50 | 154 | 5700 | 27.02 | 1.50 (1.20-1.87) | 0.0003 | 1.45 (1.15-1.83) | 0.001 |
| Oral systemic corticosteroid | 1185 | 88102 | 13.45 | 1009 | 52689 | 19.15 | 1.58 (1.44-1.72) | <0.0001 | 1.54 (1.41-1.68) | <0.0001 |
| Methylxanthine | 1151 | 85894 | 13.40 | 992 | 51513 | 19.26 | 1.59 (1.46-1.74) | <0.0001 | 1.55 (1.42-1.70) | <0.0001 |
| **Antidiabetic drugs** | | | | | | | | | | |
| Metformin | 1074 | 88320 | 12.16 | 959 | 52999 | 18.09 | 1.66 (1.52-1.82) | <0.0001 | 1.61 (1.47-1.77) | <0.0001 |
| Sulfonylureas | 1112 | 86537 | 12.85 | 979 | 52050 | 18.81 | 1.63 (1.49-1.78) | <0.0001 | 1.57 (1.43-1.72) | <0.0001 |
| DPP4-inhibitors | 174 | 39083 | 4.45 | 210 | 23565 | 8.91 | 2.71 (2.19-3.35) | <0.0001 | 2.39 (1.92-2.98) | <0.0001 |
| Insulin | 628 | 40072 | 15.67 | 594 | 26530 | 22.39 | 1.62 (1.44-1.82) | <0.0001 | 1.63 (1.44-1.83) | <0.0001 |
| **Cardiovascular drugs** | | | | | | | | | | |
| ACEI/ARB | 744 | 65925 | 11.29 | 678 | 40571 | 16.71 | 1.72 (1.55-1.92) | <0.0001 | 1.56 (1.40-1.74) | <0.0001 |
| β-blockers | 403 | 41263 | 9.77 | 381 | 25234 | 15.10 | 1.88 (1.63-2.18) | <0.0001 | 1.77 (1.52-2.05) | <0.0001 |
| Calcium-channel blockers | 678 | 55243 | 12.27 | 605 | 34303 | 17.64 | 1.69 (1.51-1.90) | <0.0001 | 1.61 (1.43-1.81) | <0.0001 |
| Diuretics | 376 | 29412 | 12.78 | 365 | 19088 | 19.12 | 1.78 (1.53-2.06) | <0.0001 | 1.71 (1.46-2.00) | <0.0001 |
| Statin | 515 | 61144 | 8.42 | 515 | 37419 | 13.76 | 1.97 (1.74-2.24) | <0.0001 | 1.72 (1.51-1.96) | <0.0001 |
| Aspirin | 486 | 43429 | 11.19 | 491 | 27272 | 18.00 | 1.86 (1.63-2.12) | <0.0001 | 1.68 (1.47-1.91) | <0.0001 |

TZD, Thiazolidinedione; PY, person-years; IR, incidence rate, per 1000 person-years; HR, hazard ratio; CAD, coronary artery disease; PAOD, peripheral arterial occlusion disease; COPD, chronic obstructive pulmonary disease; DPP-4 inhibitors, dipeptidyl peptidase-4 inhibitors; ACEI, angiotensin-converting enzyme inhibitor; ARB, angiotensin receptor blocker; DCSI, Diabetes Complications Severity Index. HR adjusted for sex, age, obesity, comorbidities, CCI score, DCSI score, and medication use.

**Supplementary Table 3.** Hazard ratios and 95% CIs of lung cancer associated with thiazolidinedione use and nonuse stratified by age, sex, comorbidities, and medications.

| **Variable** | **TZD** | | | | | | **Crude HR (95%CI)** | **p-value** | **Adjusted HR (95%CI)** | **p-value** |
| --- | --- | --- | --- | --- | --- | --- | --- | --- | --- | --- |
|  | **No** | | | **Yes** | | |  |  |  |  |
|  | **Event** | **Person Year** | **IR** | **Event** | **Person Year** | **IR** |  |  |  |  |
| Overall | 127 | 95380 | 1.33 | 125 | 57521 | 2.17 | 1.76 (1.36-2.28) | <0.0001 | 1.71 (1.32-2.22) | <0.0001 |
| Rosiglitazone | 127 | 95380 | 1.33 | 69 | 31323 | 2.20 | 1.73 (1.28-2.31) | 0.0003 | 1.65 (1.22-2.24) | 0.001 |
| Pioglitazone | 127 | 95380 | 1.33 | 56 | 26198 | 2.14 | 1.88 (1.35-2.64) | 0.0002 | 1.03 (1.01-1.05) | 0.001 |
| **Gender** | | | | | | | | | | |
| Female | 41 | 43908 | 0.93 | 27 | 26188 | 1.03 | 1.17 (0.71-1.94) | 0.52 | 1.18 (0.70-1.97) | 0.51 |
| Male | 86 | 51472 | 1.67 | 98 | 31333 | 3.13 | 2.03 (1.50-2.73) | <0.0001 | 1.96 (1.45-2.65) | <0.0001 |
| Age |  |  |  |  |  |  |  |  |  |  |
| 40-64 | 70 | 70242 | 1.00 | 54 | 36759 | 1.47 | 1.56 (1.08-2.25) | 0.01 | 1.61 (1.11-2.32) | 0.01 |
| ≧65 | 57 | 25138 | 2.27 | 71 | 20762 | 3.42 | 1.71 (1.19-2.46) | 0.003 | 1.91 (1.32-2.76) | 0.0005 |
| **Comorbidity** | | | | | | | | | | |
| CAD | 53 | 34837 | 1.52 | 52 | 21246 | 2.45 | 1.79 (1.20-2.66) | 0.004 | 1.79 (1.19-2.69) | 0.004 |
| Stroke | 28 | 17112 | 1.64 | 36 | 10725 | 3.36 | 2.20 (1.32-3.66) | 0.002 | 2.37 (1.40-4.01) | 0.001 |
| Heart failure | 16 | 6050 | 2.64 | 9 | 3747 | 2.40 | 0.94 (0.41-2.17) | 0.89 | 0.78 (0.32-1.87) | 0.57 |
| PAOD | 7 | 7332 | 0.95 | 7 | 4585 | 1.53 | 1.65 (0.57-4.78) | 0.34 | 2.30 (0.69-7.68) | 0.17 |
| Atrial fibrillation | 1 | 1384 | 0.72 | 1 | 973 | 1.03 | 2.30 (0.11-44.6) | 0.58 | -- | -- |
| Charlson comorbidity index |  |  |  |  |  |  |  |  |  |  |
| 0 | 81 | 66894 | 1.21 | 70 | 34038 | 2.06 | 1.78 (1.28-2.48) | 0.0005 | 1.70 (1.22-2.38) | 0.001 |
| 1 | 17 | 13441 | 1.26 | 23 | 10686 | 2.15 | 2.26 (1.15-4.46) | 0.01 | 2.40 (1.20-4.80) | 0.01 |
| ≧2 | 29 | 15045 | 1.93 | 32 | 12797 | 2.50 | 1.37 (0.82-2.30) | 0.22 | 1.33 (0.78-2.27) | 0.28 |
| **Moderate exacerbation of COPD** | | | | | | | | | | |
| 0-1/previous year | 117 | 79781 | 1.47 | 117 | 47690 | 2.45 | 1.77 (1.36-2.31) | <0.0001 | 1.75 (1.33-2.28) | <0.0001 |
| ≧2/previous year | 10 | 15599 | 0.64 | 8 | 9831 | 0.81 | 1.79 (0.66-4.80) | 0.24 | 1.40 (0.49-3.94) | 0.52 |
| **Severe exacerbation of COPD** |  |  |  |  |  |  |  |  |  |  |
| 0 | 124 | 94808 | 1.31 | 123 | 57189 | 2.15 | 1.79 (1.38-2.32) | <0.0001 | 1.73 (1.33-2.25) | <0.0001 |
| ≧1/previous year | 3 | 572 | 5.24 | 2 | 332 | 6.02 | 0.89 (0.14-5.37) | 0.9 | -- | -- |
| **DCSI score** | | | | | | | | | | |
| 0 | 88 | 69937 | 1.26 | 95 | 42064 | 2.26 | 1.93 (1.43-2.60) | <0.0001 | 1.77 (1.30-2.40) | 0.0002 |
| 1 | 20 | 14030 | 1.43 | 8 | 8341 | 0.96 | 0.82 (0.35-1.91) | 0.64 | 0.80 (0.33-1.91) | 0.61 |
| ≧2 | 19 | 11413 | 1.66 | 22 | 7116 | 3.09 | 1.87 (1.00-3.52) | 0.05 | 2.46 (1.25-4.82) | 0.008 |
| **Respiratory drugs** | | | | | | | | | | |
| β2bronchodilators inhalants | 99 | 30970 | 3.20 | 92 | 19432 | 4.73 | 1.61 (1.20-2.16) | 0.001 | 1.59 (1.18-2.14) | 0.002 |
| Anticholinergic inhalants | 68 | 14583 | 4.66 | 64 | 9618 | 6.65 | 1.47 (1.03-2.08) | 0.02 | 1.56 (1.09-2.22) | 0.01 |
| Corticosteroid inhalants | 12 | 10197 | 1.18 | 15 | 6045 | 2.48 | 2.25 (1.02-4.96) | 0.04 | 2.31 (1.01-5.27) | 0.04 |
| Oral systemic corticosteroid | 125 | 90856 | 1.38 | 122 | 54624 | 2.23 | 1.76 (1.36-2.28) | <0.0001 | 1.70 (1.31-2.21) | <0.0001 |
| Methylxanthine | 122 | 88583 | 1.38 | 122 | 53449 | 2.28 | 1.80 (1.38-2.33) | <0.0001 | 1.74 (1.34-2.27) | <0.0001 |
| **Antidiabetic drugs** | | | | | | | | | | |
| Metformin | 115 | 90698 | 1.27 | 117 | 54867 | 2.13 | 1.81 (1.38-2.36) | <0.0001 | 1.76 (1.34-2.30) | <0.0001 |
| Sulfonylureas | 118 | 89042 | 1.33 | 115 | 53965 | 2.13 | 1.75 (1.34-2.28) | <0.0001 | 1.70 (1.29-2.22) | 0.0001 |
| DPP4-inhibitors | 22 | 39258 | 0.56 | 23 | 23786 | 0.97 | 2.53 (1.36-4.72) | 0.003 | 2.24 (1.18-4.25) | 0.01 |
| Insulin | 55 | 41217 | 1.33 | 58 | 27506 | 2.11 | 1.83 (1.25-2.69) | 0.001 | 1.93 (1.30-2.87) | 0.001 |
| **Cardiovascular drugs** | | | | | | | | | | |
| ACEI/ARB | 82 | 67537 | 1.21 | 83 | 41815 | 1.98 | 1.86 (1.35-2.56) | 0.0001 | 1.69 (1.22-2.34) | 0.001 |
| β-blockers | 37 | 42238 | 0.88 | 54 | 25948 | 2.08 | 2.78 (1.79-4.30) | <0.0001 | 2.59 (1.65-4.05) | <0.0001 |
| Calcium-channel blockers | 73 | 56691 | 1.29 | 71 | 35369 | 2.01 | 1.76 (1.25-2.47) | 0.001 | 1.74 (1.23-2.45) | 0.001 |
| Diuretics | 38 | 30131 | 1.26 | 42 | 19735 | 2.13 | 1.86 (1.18-2.93) | 0.006 | 1.86 (1.16-2.98) | 0.009 |
| Statin | 55 | 62230 | 0.88 | 65 | 38430 | 1.69 | 2.27 (1.56-3.31) | <0.0001 | 1.90 (1.29-2.79) | 0.001 |
| Aspirin | 48 | 44457 | 1.08 | 59 | 28184 | 2.09 | 2.32 (1.55-3.47) | <0.0001 | 2.07 (1.37-3.11) | 0.0005 |

TZD, Thiazolidinedione; PY, person-years; IR, incidence rate, per 1000 person-years; HR, hazard ratio; CAD, coronary artery disease; PAOD, peripheral arterial occlusion disease; COPD, chronic obstructive pulmonary disease; DPP-4 inhibitors, dipeptidyl peptidase-4 inhibitors; ACEI, angiotensin-converting enzyme inhibitor; ARB, angiotensin receptor blocker; DCSI, Diabetes Complications Severity Index. HR adjusted for sex, age, obesity, comorbidities, CCI score, DCSI score, and medication use.

**Supplementary Table 4.** Hazard ratios and 95% CI of major cardiovascular diseases associated with different glitazones.

| **Variable** | **Stroke** | | | | | | | **Crude HR (95%CI)** | **p-value** | **Adjusted HR (95%CI)** | **p-value** |
| --- | --- | --- | --- | --- | --- | --- | --- | --- | --- | --- | --- |
|  | **TZD no-use** | | |  | **TZD use** | | |  |  |  |  |
|  | **Event** | **Person year** | **IR** |  | **Event** | **Person year** | **IR** |  |  |  |  |
| **Overall** | 101 | 95114 | 1.06 |  | 96 | 57334 | 1.67 | 1.63 (1.23-2.18) | 0.0007 | 1.63 (1.21-2.18) | 0.001 |
| **Rosiglitazone** | 101 | 95114 | 1.06 |  | 77 | 31127 | 2.47 | 2.29(1.70-3.09) | <0.0001 | 2.46(1.82-3.33) | <0.0001 |
| **Pioglitazone** | 101 | 95114 | 1.06 |  | 19 | 26207 | 0.72 | 0.72(0.43-1.19) | 0.20 | 0.65(0.39-1.07) | 0.09 |
| **Variable** | **Coronary artery disease** | | | | | | | **Crude HR (95%CI)** | **p-value** | **Adjusted HR (95%CI)** | **p-value** |
|  | **TZD no-use** | | |  | **TZD use** | | |  |  |  |  |
|  | **Event** | **Person year** | **IR** |  | **Event** | **Person year** | **IR** |  |  |  |  |
| **Overall** | 98 | 95116 | 1.03 |  | 88 | 57361 | 1.53 | 1.54 (1.14-2.07) | 0.004 | 1.55 (1.15-2.10) | 0.003 |
| **Rosiglitazone** | 98 | 95116 | 1.03 |  | 70 | 31143 | 2.25 | 2.16(1.59-2.95) | <0.0001 | 2.37(1.73-3.25) | <0.0001 |
| **Pioglitazone** | 98 | 95116 | 1.03 |  | 18 | 26218 | 0.69 | 0.69(0.41-1.16) | 0.16 | 0.63(0.37-1.05) | 0.08 |
| **Variable** | **Heart failure** | | | | | | | **Crude HR (95%CI)** | **p-value** | **Adjusted HR (95%CI)** | **p-value** |
|  | **TZD no-use** | | |  | **TZD use** | | |  |  |  |  |
|  | **Event** | **Person year** | **IR** |  | **Event** | **Person year** | **IR** |  |  |  |  |
| **Overall** | 52 | 95358 | 0.55 |  | 44 | 57556 | 0.76 | 1.60 (1.06-2.43) | 0.02 | 1.61 (1.06-2.46) | 0.02 |
| **Rosiglitazone** | 52 | 95358 | 0.55 |  | 35 | 31319 | 1.12 | 2.12(1.37-3.27) | <0.0001 | 2.24(1.45-2.48) | 0.0003 |
| **Pioglitazone** | 52 | 95358 | 0.55 |  | 9 | 26237 | 0.34 | 0.75(0.36-1.57) | 0.45 | 0.72(0.35-1.50) | 0.38 |

**Supplementary Table 5.** Cox model measured hazard ratios and 95% confidence interval of bacterial pneumonia associated with defined daily dose and prescribed daily dose.

| **Matched cohort** | **Bacterial pneumonia** | | | **Crude** | | **Adjusted** | |
| --- | --- | --- | --- | --- | --- | --- | --- |
|  | **Event** | **Person-year** | **Incidence** | **HR (95% CI)** | ***p* value** | **HR (95% CI)** | ***p* value** |
| Non-users (n=12856) | 1205 | 92596 | 13.01 | **1 (reference)** | | **1 (reference)** | |
| Users (n=12856) | 1040 | 55505 | 18.74 | 1.59 (1.46-1.74) | <0.0001 | 1.55 (1.42-1.70) | <0.0001 |
| **Cumulative duration of therapy (days)** | | |  |  |  |  |  |
| Non-use | 1205 | 92596 | 13.01 | **1 (reference)** | | **1 (reference)** | |
| <100 | 186 | 7762 | 23.96 | 1.97(1.68-2.30) | <0.0001 | 1.55(1.32-1.81) | <0.0001 |
| 100-300 | 153 | 7333 | 20.86 | 1.71(1.44-2.03) | <0.0001 | 1.63(1.37-1.93) | <0.0001 |
| >300 | 701 | 40410 | 17.35 | 1.50(1.36-1.65) | <0.0001 | 1.54(1.40-1.71) | <0.0001 |
| **Cumulative defined daily doses of TZD therapy (DDD/month)** | | | |  |  |  |  |
| Non-use | 1205 | 92596 | 13.01 | **1 (reference)** | | **1 (reference)** | |
| <5 | 257 | 11382 | 22.58 | 1.86(1.62-2.13) | <0.0001 | 1.52(1.32-1.75) | <0.0001 |
| 5-10 | 103 | 4880 | 21.11 | 1.71(1.40-2.10) | <0.0001 | 1.73(1.41-2.11) | <0.0001 |
| >10 | 680 | 39243 | 17.33 | 1.50(1.35-1.65) | <0.0001 | 1.54(1.40-1.71) | <0.0001 |

PY, person-years; Incidence rate, per 1000 person-years; HR, hazard ratio; CI, confidence interval. Models adjusted by gender, age, body mass index, comorbidities, Charlson comorbidity index, moderate exacerbation of COPD, severe exacerbation of COPD, DCSI score, and medications listed in Table 1.

**Supplementary Table 6.** Cox model measured hazard ratios and 95% confidence interval of IMV associated with defined daily dose and prescribed daily dose.

| **Matched cohort** | **IMV** | | | | **Crude** | | **Adjusted** | |
| --- | --- | --- | --- | --- | --- | --- | --- | --- |
|  | **Event** | **Person-year** | | **Incidence** | **HR (95% CI)** | ***p* value** | **HR (95% CI)** | ***p* value** |
| Non-users (n=12856) | 762 | 94673 | | 8.05 | **1 (reference)** | | **1 (reference)** | |
| Users (n=12856) | 556 | 57112 | | 9.74 | 1.33 (1.19-1.49) | <0.0001 | 1.23 (1.09-1.37) | 0.0004 |
| **Cumulative duration of therapy (days)** | | |  | |  |  |  |  |
| Non-use | 762 | 94673 | | 8.05 | **1 (reference)** | | **1 (reference)** | |
| <100 | 128 | 8039 | | 15.92 | 2.11(1.75-2.55) | <0.0001 | 1.54(1.28-1.87) | <0.0001 |
| 100-300 | 82 | 7588 | | 10.81 | 1.43(1.14-1.80) | 0.002 | 1.19(0.94-1.49) | 0.14 |
| >300 | 346 | 41485 | | 8.34 | 1.15(1.10-1.31) | 0.03 | 1.14(1.01-1.31) | 0.04 |
| **Cumulative defined daily doses of TZD therapy (DDD/month)** | | | | |  |  |  |  |
| Non-use | 762 | 94673 | | 8.05 | **1 (reference)** | | **1 (reference)** | |
| <5 | 169 | 11774 | | 14.35 | 1.90(1.61-2.25) | <0.0001 | 1.44(1.21-1.70) | <0.0001 |
| 5-10 | 51 | 5059 | | 10.08 | 1.32(1.00-1.75) | 0.05 | 1.28(0.96-1.70) | 0.09 |
| >10 | 336 | 40279 | | 8.34 | 1.16(1.02-1.32) | 0.03 | 1.14(1.00-1.30) | 0.05 |

PY, person-years; Incidence rate, per 1000 person-years; HR, hazard ratio; CI, confidence interval. Models adjusted by gender, age, body mass index, comorbidities, Charlson comorbidity index, moderate exacerbation of COPD, severe exacerbation of COPD, DCSI score, and medications listed in Table 1.

**Supplementary Table 7.** Cox model measured hazard ratios and 95% confidence interval of lung cancer associated with defined daily dose and prescribed daily dose.

| **Matched cohort** | **Lung cancer** | | | **Crude** | | **Adjusted** | |
| --- | --- | --- | --- | --- | --- | --- | --- |
|  | **Event** | **Person-year** | **Incidence** | **HR (95% CI)** | ***p* value** | **HR (95% CI)** | ***p* value** |
| Non-users (n=12856) | 127 | 95380 | 1.33 | **1 (reference)** | | **1 (reference)** | |
| Users (n=12856) | 125 | 57521 | 2.17 | 1.76 (1.36-2.28) | <0.0001 | 1.71 (1.32-2.22) | <0.0001 |
| **Cumulative duration of therapy (days)** | | |  |  |  |  |  |
| Non-use | 127 | 95380 | 1.33 | **1 (reference)** | | **1 (reference)** | |
| <100 | 18 | 8147 | 2.21 | 1.73(1.05-2.85) | 0.03 | 1.33(0.81-2.20) | 0.25 |
| 100-300 | 19 | 7647 | 2.48 | 1.94(1.20-3.16) | 0.007 | 1.80(1.10-2.94) | 0.02 |
| >300 | 88 | 41727 | 2.11 | 1.73(1.31-2.30) | 0.0001 | 1.80(1.35-2.40) | <0.0001 |
| **Cumulative defined daily doses of TZD therapy (DDD/month)** | | | |  |  |  |  |
| Non-use | 127 | 95380 | 1.33 | **1 (reference)** | | **1 (reference)** | |
| <5 | 27 | 11919 | 2.27 | 1.78(1.17-2.71) | 0.006 | 1.41(0.92-2.14) | 0.11 |
| 5-10 | 14 | 5096 | 2.75 | 2.13(1.23-3.72) | 0.007 | 2.13(1.22-3.72) | 0.007 |
| >10 | 84 | 40506 | 2.07 | 1.71(1.29-2.27) | 0.0002 | 1.78(1.34-2.38) | <0.0001 |

PY, person-years; Incidence rate, per 1000 person-years; HR, hazard ratio; CI, confidence interval. Models adjusted by gender, age, body mass index, comorbidities, Charlson comorbidity index, moderate exacerbation of COPD, severe exacerbation of COPD, DCSI score, and medications listed in Table 1.

**Supplementary Table 8.** Outcomes of thiazolidinedione users and nonusers (sensitivity test).

|  | **TZD non-users** | | | **TZD users** | | | **Crude** | | **Adjusted** | |
| --- | --- | --- | --- | --- | --- | --- | --- | --- | --- | --- |
|  | **Events** | **PY** | **IR** | **Events** | **PY** | **IR** | **HR (95% CI)** | ***p* value** | **HR (95% CI)** | ***p* value** |
| **Death** | 20 | 85432 | 0.23 | 16 | 54301 | 0.29 | 1.43(0.72-2.83) | 0.31 | 1.25(0.62-2.50) | 0.53 |
| **MACE** | 103 | 84853 | 1.21 | 73 | 53956 | 1.35 | 1.15(0.81-1.50) | 0.22 | 0.99(0.75-1.26) | 0.46 |
| Stroke | 103 | 85005 | 1.21 | 73 | 54046 | 1.35 | 1.23(0.91-1.67) | 0.18 | 1.18(0.87-1.61) | 0.29 |
| Ischemic heart disease | 101 | 85004 | 1.19 | 66 | 54051 | 1.22 | 1.11(0.80-1.52) | 0.53 | 1.09(0.79-.50) | 0.62 |
| Heart failure | 57 | 85252 | 0.67 | 38 | 54519 | 0.70 | 1.05(0.66-1.65) | 0.83 | 1.04(0.65-1.64) | 0.87 |
| **Hospitalization for COPD** | 576 | 83753 | 6.88 | 412 | 53360 | 7.72 | 1.21(1.06-1.38) | 0.005 | 1.05(0.92-1.19) | 0.51 |
| **NIPPV** | 191 | 85179 | 2.24 | 169 | 54059 | 3.13 | 1.66(1.34-2.07) | <0.0001 | 1.49(1.20-1.86) | 0.0003 |
| **IMV** | 700 | 84595 | 8.27 | 519 | 53737 | 9.66 | 1.32(1.17-1.48) | <0.0001 | 1.17(1.05-1.32) | 0.007 |
| **Bacterial pneumonia** | 1088 | 82871 | 13.13 | 995 | 52160 | 19.08 | 1.65(1.51-1.81) | <0.0001 | 1.58(1.44-1.73) | <0.0001 |
| **Lung cancer** | 119 | 85250 | 1.40 | 113 | 54150 | 2.09 | 1.67(1.28-2.18) | 0.0002 | 1.56(1.19-2.05) | 0.001 |

TZD, thiazolidinedione; PY, person-years; IR, incidence rate, per 1000 person-years; HR, hazard ratio; MACE, major adverse cardiovascular events; COPD, chronic obstructive pulmonary disease; NIPPV, noninvasive positive pressure ventilation; IMV, invasive mechanical ventilation. ^*^Models adjusted by sex, age, obesity, smoking status, Charlson Comorbidity Index, moderate exacerbation of COPD, severe exacerbation of COPD, DCSI score, DM duration, and medications listed in Table 1.
